# Exploring the Influence of Failure Aversion on Learning From Project Failure: A Sensemaking Perspective

**DOI:** 10.3389/fpsyg.2022.794390

**Published:** 2022-05-03

**Authors:** Liangting Zhang, Bin Wang, Xiaoxia Feng, Yue Zhang, Wenzhou Wang

**Affiliations:** ^1^Management School, Hainan University, Haikou, China; ^2^School of Management, Shanghai University, Shanghai, China; ^3^Business School, Beijing Normal University, Beijing, China

**Keywords:** learning from project failure, sensemaking, failure aversion, loss of self-esteem, loss-focused coping, learning goal orientation

## Abstract

Although project failure is commonly considered a negative event, it can provide valuable resources for learning. Despite well documented research on the antecedents of learning from project failure at the individual level, individuals’ attitude toward failures, a relatively proximal antecedent of learning from failure, has attracted limited attention in organizational studies. To address this paucity of research, based on the sensemaking theory, in the current study we specifically focused on individuals’ failure aversion and explored how it would influence learning from failure through the process of arguing and expectation. Using a sample of 774 employees from R&D teams in China, our findings revealed that individuals’ failure aversion enhanced their learning from failure through inducing a loss-focused coping, but failure aversion negatively affected learning from failure through increasing the individuals’ perceived loss of self-esteem. We also found that individuals’ learning goal orientation (LGO) weakened the negative relationship between the loss of self-esteem and learning from failure; however, LGO did not moderate our hypothesized relationship between loss-focused coping and learning from failure. Our study extends the literature on learning from failure in two ways. First, it explores the learning from failure process at the individual level based on the sensemaking theory and second, it sheds light on the underlying cognitive mechanisms operating between failure aversion and learning from project failure.

## Introduction

Project failure is common and sometimes inevitable in the current fast-changing world, which hurts not only the initiative of organizations or individuals but also their reputations. Besides, organizations and individuals suffering from failure may also face financial losses and stress (Dahlin et al., 2018; Patzelt et al., 2021). Despite these negative effects, failure could also be seen as a resource which enables organizations and individuals to learn, thereby making changes and preventing future failures. The potential value of failure has been acknowledged (e.g., Shepherd et al., 2011; Josefy et al., 2017), and the benefits of learning from failure are well documented. These include innovation (Jiménez-Jiménez and Sanz-Valle, 2011; Danneels and Vestal, 2020), better financial performance (Hirak et al., 2012), and more knowledge creation (Phan and Peridis, 2000). However, despite the potential positive effects generated by learning from failure, organizations or individuals may not necessarily take the initiative (i.e., to learn) after failure (Shepherd et al., 2011), which encourages researchers to explore the antecedents of learning from failure.

Previous researchers have identified a set of predictors for learning from failure, such as the organizational culture/climate (Van Dyck et al., 2005), organizational leadership (Ye et al., 2019), and individuals’ personalities (Zhao, 2011). However, individual’s attitude toward failures (Frese and Keith, 2015), a relatively proximal antecedent of learning from failure, has attracted limited attention in the existing management literature. Yet, if we look further afield to other disciplines, studies in educational psychology and behavioral economics have revealed the crucial role of individuals’ general attitudes toward failures in influencing their learning behaviors after failures. For instance, Alamri and Fawzi (2016) found that students with a positive attitude toward making oral errors (where failure is theorized as a specific type of error) showed a better language learning performance.

To address this theoretical gap, in the current study we aim to examine how individuals’ attitudes would influence their learning behaviors after failure in the work context, based on a sensemaking perspective. Sensemaking, as the name implies, refers to the making of sense (Weick, 1995), that is, active agents’ construction of the meaning of interrupted events (Huber and Daft, 1987). The failure is seen as an interruption to an ongoing event that individuals expected to be a success. The central question for sensemaking is to explore how, why, and with what effects agents construct the meaning of the interrupted events (Weick, 1995). Previous research has identified that problem definition, perceived environmental uncertainty, and conditions for conscious cognitive processing can all seed sensemaking (Weick, 1995). Project failure can be seen as a problem. The problem refers to “some kind of gap, difference, or disparity between the way things are and the way one wants them to be” (Smith, 1988). Project failure will lead employees to perceive a discrepancy from the predicted success. This perceived discrepancy, functioning as a clue, will trigger individuals to explain it, and then take actions (e.g., learning from failure) to respond to it. In this process, sensemaking is developed through a sequence of steps occurring over time, which provides us with a framework to explain how and why individuals exhibit learning from failure when they experience project failure.

Individuals’ attitude toward failure plays a crucial role in the sensemaking process because it makes individuals interpret failure events from different perspectives. Failure always leads to losses, thus people are likely to be disappointed with failures and take negative attitudes toward them, that is, they experience failure aversion (Yechiam and Hochman, 2013, 2014). When individuals notice failure events, they began to construct meanings for these failure events by a process of arguing and considering expectations (Weick, 1995). Arguing refers to the process of collision and fusion between positive and negative cognitions triggered by the attitudes toward failure (i.e., failure aversion). This process explains how individuals construct meaning by using a variety of cognitions. On the other hand, expecting refers to anticipation or foresight that can guide interpretations. If the outputs conform to what was expected, individuals will not notice any discrepancy; only when the outputs violate the expectation, individuals will perceive a discrepancy, leading to corresponding behavioral responses to fill the gap.

For the arguing process, individuals will generate different cognitions (including positive and negative aspects), which exert different effects on the process of learning from failure. When failure is framed as a negative or stigmatized event, people tend to evaluate themselves negatively (e.g., they experience self-doubt and self-worthlessness), devote their cognition to understand encountered failure, and find solutions to mitigate against its repetition (Frese and Keith, 2015). Building upon this rationale, we therefore propose that individuals with higher failure aversion are more likely to (1) hold a more negative self-evaluation after failure (i.e., report more loss of self-esteem) and (2) allocate more cognitive resources to the failure event (i.e., more frequently show a loss-focused coping). That is to say, according to the arguing process, failure aversion may trigger two inconsistent cognitive tactics in individuals (positive and negative): a loss of self-esteem and a loss-focused form of coping. By using various cognitive tactics to interpret failure events, people then decide what actions they may take to respond to this failure event. These decisions may hinder or promote individual learning from failure. As mentioned earlier, the loss of self-esteem and loss-focused coping, as arguing processes both triggered by failure aversion, will further influence individual learning from failure. We expect that the loss of self-esteem will hurt individuals’ motivation to learn and therefore negatively effect on learning from failure; loss-focused coping, on the other hand, can help people allocate their cognitive resources to the failed event and will be conducive to learning from failure.

In addition to the arguing process, expecting is another important sensemaking process to influence individual learning from failure. Unlike arguing that involves the general process of sensemaking, considering expectations mainly focuses on individual differences because different individuals have different expectations toward failure, which will lead to different behavioral responses. After suffering from failure, learning from failure is one of the tactics that can help individuals alleviate the negative impact of failure, yet the degrees of expectations toward learning from failure are different between people. In this study, we mainly concentrate on learning goal orientation (LGO) which is an important factor that determines the individuals’ expectation toward learning from failure. Individuals with higher levels of LGO have stronger desires to develop themselves by acquiring new skills, mastering new situations (i.e., recovering from failure), and improving their competence (Noordzij et al., 2013; Matsuo, 2021). They have higher expectations concerning learning from failure and making success. However, individuals with lower levels of LGO are unwilling to accept new knowledge and master new situations so that they will have lower expectations about learning from failure. Despite the important role of LGO, the direct effect of LGO on learning from failure was not supported in the work context (Zhao, 2011). Thus, we argue that LGO may not matter directly; instead, LGO may influence learning from failure through weakening or strengthening the arguing process of the loss of self-esteem and a loss-focused coping on learning activities. Specifically, individuals with a higher LGO regard unexpected situations (i.e., failure context) as a source of knowledge and they enjoy mastery over challenging or stressful environments (Vandewalle, 1997). Thus, individuals with a higher LGO can promote their own motivation to learn when they experience the loss of self-esteem because such individuals have a desire to develop their competence by learning and they also believe they can learn. As a result, they are more likely to find the value of learning from failure and they develop their self-esteem needs by learning. This, in turn, weakens the negative effect of the loss of self-esteem on learning from failure. Thus, when they encounter a project failure, they will allocate more cognitive resources to cope with that failure as well as experiencing a set of negative emotions caused by the failure event, which in turn accentuates the positive relationship between loss-focused coping and learning from failure.

**FIGURE 1:**
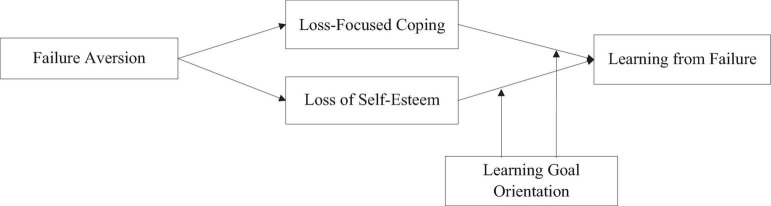
Theoretical model.

In conclusion, based on the sensemaking theory, we aim to explore how failure aversion could influence individuals’ learning from failure through the effect of experiencing a failure event on their cognitive processes (i.e., the loss of self-esteem and loss-focused coping) and we examine the moderating effect of LGO in the relationship between cognitive responses and learning from failure. The theoretical model was shown in Figure 1.

Our study makes contributions in three ways. First, we extend the literature on the antecedents of learning from failure by hypothesizing about and examining individuals’ attitudes toward failure (i.e., failure aversion) based on the sensemaking theory. Second, and most importantly, we propose two cognitive pathways as arguments to link failure aversion and learning from failure, addressing both the bright and dark sides of failure aversion. Finally, we reveal the moderating role of the LGO (as an expectation) in the relationship between cognitive reactions and learning from failure.

## Hypothesis Development

### The Mediating Role of the Loss of Self-Esteem

Considering the relationship between failure aversion and loss of self-esteem, we expect that failure aversion have positive effect on loss of self-esteem. As mentioned earlier, individuals’ attitude toward failure will influence their cognitive responses (e.g., self-evaluations) after failure. Self-esteem indicates an individual’s overall self-evaluation of his/her competencies (Pierce and Gardner, 2004). Individuals’ feelings of efficacy and competence induced by their personal experiences play a crucial role in influencing their self-esteem (Pierce and Gardner, 2004). Generally speaking, successful experiences will increase one’s self-esteem, while one is more likely to perceive a loss of self-esteem after failure. Moreover, the individuals’ attitude toward failure will also affect individuals’ interpretations of experiencing a failure event. Bandura (1997) has pointed out that individuals’ interpretations of their performance (e.g., failure or success) play a deeper role in influencing self-esteem after a specific event. For instance, individuals with lower levels of failure aversion tend to regard project failure as a common event in the workplace, and they may not necessarily report a loss of self-esteem after failure.

In contrast, according to Crocker and Wolfe (2001), individuals’ self-esteem is based on their experience in specific domains on which they have staked their self-worth. When they experience failure in the domains where they have staked their self-worth, they will experience a loss of self-esteem (Jenkins et al., 2014). Individuals with higher levels of failure aversion tend to stake their self-worth on the project and interpret encountered failure as a sign of personal incompetence. Thus, they may experience more self-worthlessness and self-doubt after failure, which in turn triggers a greater loss of their own self-esteem. We therefore propose that:

*Hypothesis 1: Failure aversion is positively associated with the loss of self-esteem.*

When individuals construct the meaning of failure to be associated with negative cognition (i.e., loss of self-esteem), we expect that this negative cognition will impede individuals’ motivation to learn and therefore this has a negative effect on learning from failure. Learning from failure refers to “the sense that one is acquiring, and can apply, knowledge and skills” (Shepherd et al., 2011). As a self-regulated behavior, learning from failure is driven by individuals’ motivation (i.e., desire or willingness) to participate in learning from failure activities (Dahlin et al., 2018). The motivation to learn depends on individuals’ resource levels that can be devoted to learning activities (Dahlin et al., 2018), the perceived value of learning, and the probability of achieving the learning outcome (Zhao, 2011). Building upon the literature on the impact of self-evaluation on motivation and behavior (e.g., Boekaerts, 1996; Erez and Judge, 2001) which emphasizes the crucial role of self-evaluation in determining individual motivation and behaviors, we posit that the loss of self-esteem will reduce individuals’ motivation to learn from failure by redirecting their cognitive resources and distorting their expectations and valence of learning from failure.

More specifically, when people experience the loss of self-esteem, they will redirect their attention to their personal needs (i.e., maintaining and enhancing their self-esteem, Tharenou (1979) to sustain the desired self-esteem state rather than engaging in learning activities. Given that individuals only have limited resources (Hobfoll, 1989; Hobfoll et al., 2018), these people who allocate their cognitive resources to maintain self-esteem may not have enough personal resources to find useful information and learn from their failures.

In addition, the loss of self-esteem will also reduce individuals’ motivation to learn through reducing their perceived expectations and the valence of their learning. Individuals who experience a loss of self-esteem may treat the temporary failure as permanent. In this situation, individuals will perceive learning to have a lower valence because the failure seems unsolvable. Moreover, individuals who experience the loss of self-esteem will feel worthless (Jenkins et al., 2014). Thus they will not trust their capabilities to cope with failures, which may lower their perceptions of the probability of their achieving the learning outcomes. Although the link between self-evaluation and learning behavior has not been examined directly, one researcher has posited that individuals with lower personal value (the core content of self-esteem) would have lower expectations and valence beliefs (Feather, 1988). Meanwhile, researchers have also pointed out that individuals’ expectations and valence judgments are positively associated with their motivation to learn (Zhao, 2011).

In sum, employees who report more loss of self-esteem after a failure at work are more likely to exert their efforts to maintain or enhance their self-esteem and, as well, they tend to perceive learning to have a lower value, resulting in a lower level of learning from failure. Hence, we propose that:

*Hypothesis 2: The loss of self-esteem is negatively associated with learning from failure.*

In conclusion, based on the sensemaking theory, we argue that individuals high in failure aversion interpret failure in a negative manner and experience more loss of self-esteem after failure, which in turn reduces their learning from failure:

*Hypothesis 3: The loss of self-esteem mediates the relationship between failure aversion and learning from failure.*

### The Mediating Role of Loss-Focused Coping

In addition to negative cognition tactics, we also expect that failure aversion can trigger individuals’ positive cognition tactics to understand the encountered failure and find solutions to it, i.e., to engage in loss-focused coping. Loss-focused coping refers to an “individual’s efforts to work through and process aspects of a loss to break the emotional bonds to the object lost” (Shepherd et al., 2011). In fact, the strategies to cope with failure could vary from individual to individual (Shepherd, 2003). We argue that individuals high in failure aversion adopt loss-focused coping to deal with their encountered failure for several reasons.

First, individuals with higher levels of failure aversion tend to frame failure as a stigmatizing event (Van Dyck et al., 2005). In order to restore their reputation, they will try to control and minimize the influence of the failure event by adding more attention into the failure events and processing related information to work through the problems associated with it (i.e., adopting loss-focused coping). The indirect evidence of this argument comes from the loss aversion literature (failure aversion can be seen as a kind of loss aversion) which has shown that individuals increase their attentional resources to cope with losses because of their loss aversion (Yechiam and Hochman, 2013, 2014).

Second, individuals with higher failure aversion may tend to experience more negative emotions such as anxiety and depression due to being afraid of committing failures. In order to deal with these negative emotions, individuals will redirect their attention to break the emotional bonds toward failures, thus improving their individual loss-focused coping. This proposition about the facilitating role of negative emotion is consistent with the argument that individuals who experience negative emotion may respond by prioritizing emotion-focused coping (Zhao, 2011). In conclusion, we expect that individuals’ failure aversion can facilitate loss-focused coping after they experience failure:

*Hypothesis 4: Failure aversion is positively associated with loss-focused coping.*

When individuals construct the meaning of failure with positive cognition (i.e., with loss-focused coping), we expect that this cognition has a positive effect on learning from failure. By adopting loss-focused coping, individuals will allocate their attentional resources to cope with failure as well as experiencing a set of negative emotions caused by the failure event. Focusing on the failure events can help individuals to uncover the reasons why the project failed by scanning and processing the information about the failure. By understanding the reasons for the failure, individuals can adjust their belief systems about how to deal with these failures and what should be done in the future, thus promoting their ability to learn from failure (Shepherd et al., 2011). In support of this argument, Kim and Miner (2007) showed that uncovering the reasons for project failure can increase individuals’ exploration of information (an important way to learn) about the actions and routes that could be taken in the future.

Apart from concentrating on the failure itself, when individuals deal with their negative emotions caused by failure, this can also increase their ability to learn from failure. That is, through dealing with the negative emotions, individuals can break the negative bonds to the failure events. By doing this, they can easily uncover the reason/s why the project failed rather than falling into this negative emotional state. Previously researchers have shown that individuals’ negative emotions impede their subsequent learning behavior (Shepherd, 2003; Zhao, 2011). Thus, dealing with such negative emotions can facilitate individuals’ learning behavior. Based on the above reasoning, we propose that individuals’ loss-focused coping can increase their subsequent learning activities:

*Hypothesis 5: Loss-focused coping is positively associated with learning from failure.*

In conclusion, as a stable attitude toward failure, individual failure aversion triggers individuals’ loss-focused coping, which in turn increases their subsequent learning behavior. Thus, we propose that loss-focused coping is a mediator in the relationship between failure aversion and learning from failure:

*Hypothesis 6: Loss-focused coping mediates the relationship between failure aversion and learning from failure.*

### The Moderating Role of Learning Goal Orientation

As mentioned earlier, in addition to the arguing process, expectations serve as strong filters to activate the sensemaking process, which also aroused our attention. In this study, we mainly concentrate on LGO which is an important factor to determine the individuals’ expectation toward learning from failure. LGO refers to “a desire to develop the self by acquiring new skills, mastering new situations, and improving one’s competence” (Vandewalle, 1997). Individuals with higher LGO have the desire to develop themselves by acquiring new skills, mastering new situations (i.e., recovering from failure), and improving their competence. They have higher expectations about learning from failure and achieving success. In comparison, individuals with lower LGO are unwilling to accept new knowledge and master new situations so that they will have lower expectations concerning learning from failure. We expect that LGO may influence learning from failure through weakening or strengthening the effects of the loss of self-esteem and loss-focused coping on learning activities.

Specifically, individuals who have suffered from the loss of self-esteem may shift their attention from the primary task or project to their self-esteem needs and underestimate the potential value of learning, thus they relate negatively to learning from failure. Individuals with higher LGO regard learning as the core content of their self-esteem needs because they desire to develop themselves by learning and believe they can learn (VandeWalle, 2001; Vandewalle et al., 2019). Thus, when individuals with higher LGO feel the loss of self-esteem, they will allocate more cognitive resources into learning activities to maintain their desirable self-esteem state. Therefore, LGO can weaken the negative relationship between the loss of self-esteem and learning from failure.

In addition, individuals with higher LGO are more likely to find the potential value of learning from failure because they always treat challenges as opportunities to enhance their knowledge and competence rather than as a threat to be avoided (Ng and Lucianetti, 2018; Vandewalle et al., 2019). Even though individuals with higher LGO feel the loss of self-esteem after failure events, they can still develop solution-oriented self-instruction to cope with failures and evaluate learning outcomes as valuable, thus raising their expectations and improving their perception of the valence of learning from failure. Based on the above reasoning, we propose that individuals’ LGO (as an expectation) can buffer the negative relationship between the loss of self-esteem and learning from a failure. Thus we hypothesize:

*Hypothesis 7: Learning goal orientation moderates the relationship between the loss of self-esteem and learning from a failure such that the negative relationship is weaker for employees with a higher level of learning goal orientation.*

As mentioned earlier, loss-focused coping can enhance learning behavior by breaking the negative cognitive associations about the failure and uncovering the reasons for the failure. Individuals’ LGO can influence the relationship between their loss-focused coping and learning from failure by regulating individual cognitive resources. Individuals with higher LGO tend to use positive thinking to help themselves snap back from negative emotions after failure because they always regard unexpected situations as a source of knowledge. Thus, they can easily use loss-focused coping to break the negative cognitive associations with the failure event, which in turn increases the opportunities to learn from failure. Additionally, individuals with higher LGO tend to master challenging situations, so that they will allocate more attentional resources to uncover the reasons why a project failed. As a result, when they use loss-focused coping to deal with failures, they will gain more attentional resources to construct an account of why the project failed, thus further facilitating their motivation to learn. Based on this reasoning, we hypothesize:

*Hypothesis 8: Learning goal orientation moderates the relationship between loss-focused coping and learning from a failure such that the positive relationship is stronger for employees with a higher level of learning goal orientation.*

## Materials and Methods

### Participants and Procedures

Our data were collected from research & development (R&D) teams in high-tech firms in Beijing because project failures commonly exist in R&D teams. We randomly selected 400 firms from the list of high-tech industries which was provided by the Beijing Municipal Science and Technology Commission. We then contacted the CEO or chairman of these selected firms to invite them to participate in our research. If the CEO or chairman agreed to participate, they wrote an endorsement letter and chose a coordinator (usually from the human resource management) to help us distribute surveys. At the firm’s weekly or monthly team meeting, we introduced the purpose of this project and assured its confidentiality to encourage employees to participate. For those who were absent from this meeting, we left our survey, informed consent information, and self-addressed stamped envelopes for them. Employees volunteered to complete the survey. The final dataset consisted of 774 participants (22.87% were female) from 58 companies. The average age was 31.73 years old and most participants (88.8%) held a Bachelor’s or Master’s degree.

### Measures

In this study, we defined project failure depending on the results of research projects. To help participants better understand project failure, in the introduction to the questionnaire we defined it as “the termination of an initiative to create organizational value that has fallen short of its goals” (Shepherd et al., 2011). In the first part, participants were asked to report their demographic information and usual attitude (i.e., to failure aversion and their LGO). They then were asked to recall a recent project failure and report their personal experiences or behaviors after this failure (i.e., loss of self-esteem, loss-focused coping, and learning from failure). All responses were measured by 6-point scales with anchors ranging from 1 = *totally disagree* to 6 = *totally agree*.

#### Failure Aversion

We measured failure aversion using five items adapted from Van Dyck et al. (2005). The items were “I feel stressed when our project failed,” “I get upset and irritated if a failure occurs,” “I feel embarrassed after suffering from failure,” “I am often afraid of experiencing failure,” and “I am often concerned that failure might occur.” The Cronbach’s alpha was 0.83.

#### Loss of Self-Esteem

We measured loss of self-esteem using five items adapted from Jenkins et al. (2014). The items were “I have lost recognition or status,” “I have lost faith in myself as a capable men/woman,” “I have lost an important person’s approval or respect,” “I feel that I have lost my good reputation,” and “I have lost my self-respect.” The Cronbach’s alpha was 0.88.

#### Loss-Focused Coping

We measured loss-focused coping using six items developed by Shepherd et al. (2011). The items were “In my mind, I often went over the events leading up to the project’s failure,” “I actively worked with others to make sense of the failure,” “I make sure I talk through my emotions about the failure with others,” “I frequently seek out people to talk about my negative feelings generated from the project’s failure,” “I confront my thoughts about the failure of the project,” and “I work through my negative emotions generated by the project’s failure.” The Cronbach’s alpha was 0.70.

#### Learning From Failure

We measured learning from failure using eight items developed by Shepherd et al. (2011). The items were “I have learned to better execute a project,” “I now realize the mistakes that we made that led to the project’s failure,” “I can more effectively run a project,” “I have improved my ability to make important contributions to a project,” “I can ‘see’ earlier the signs that a project is in trouble,” “I am more willing to help others deal with their failures,” “I am more tolerant of others’ shortcomings when it comes to projects,” and “I am a more forgiving person at work.” The Cronbach’s alpha was 0.91.

#### Learning Goal Orientation

We measured LGO using five items developed by Vandewalle (1997). Sample items were “I am willing to select a challenging work assignment that I can learn a lot from,” “I prefer to work in situations that require a high level of ability and talent,” “I often look for opportunities to develop new skills and knowledge,” “I enjoy challenging and difficult tasks at work where I will learn new skills,” and “For me, development of my work ability is important enough to take risks.” The Cronbach’s alpha was 0.85.

#### Control Variables

Following previous studies (e.g., Shepherd et al., 2011), we controlled for demographic variables such as gender (0 = male, 1 = female), age, educational background (ranging from “1 = high school” to “6 = Ph.D., or other professional degrees”), and time since the project failure.

### Analytical

To test our hypotheses, a path analysis approach was used. This has been proved to overcome some shortcomings of the causal steps approach (Hill et al., 2014). Path analysis allows us to test a dual-pathway model simultaneously, which is closely related to our theoretical model. We first regressed the mediators (i.e., loss of self-esteem, loss-focused coping) on control variables (i.e., gender, age, education level, and time since the project failure) and predictor variables (i.e., failure aversion) to test Hypotheses 1 and 4. Then we regressed the dependent variables (i.e., learning from failure) on the control variables, predictor variables, mediator variables, and their interaction, to test Hypotheses 2, 5, 7, and 8. Finally, we used a bootstrapping procedure to test the indirect effects, that is, to test Hypotheses 3 and 6.

## Results

### Validity and Common Method Variance

We first conducted a confirmatory factor analysis (CFA). As shown in Table 1, the theoretical model (χ^2^ = 1268.78, df = 364, χ^2^/df = 3.49, RMSEA = 0.06, GFI = 0.90, TLI = 0.91, CFI = 0.92) fitted better than alternative models (i.e., the four-factor, three-factor, two-factor, and one-factor models), indicating the construct distinctiveness of our study variables.

**TABLE 1:** Comparison of measurement model.

Model	Factor	χ^2^	*df*	χ^2^/df	RMSEA	GFI	CFI	TLI
Theoretical model	Five factors: ES, LS, CS, FL, LGO	1268.78	364	3.49	0.06	0.90	0.92	0.91
Model 1	Four factors: ES, LS + CS, FL, LGO	2842.08	371	7.66	0.09	0.75	0.79	0.77
Model 2	Three factors: FL ES + LS + CS, LGO	4087.29	374	10.93	0.11	0.67	0.69	0.66
Model 3	Three factors: ES, LS + CS + LGO, FL	4528.83	374	12.11	0.12	0.63	0.65	0.62
Model 4	Two factors: ES + LS + CS + LGO, FL	5774.97	376	15.36	0.14	0.58	0.54	0.51
Model 5	One factors: ES + LS + CS + LGO + FL	7095.14	377	18.82	0.15	0.53	0.43	0.39

*ES, failure aversion; LS, loss of self-esteem; CS, loss-focused coping; FL, learning from failure; LGO, learning goal orientation.*

Given the cross-sectional design of the current study, we further conducted a Harman’s single-factor analysis to examine the potential for common methods bias (CMB) (Podsakoff et al., 2003). Our results showed no general factor accounting for a majority of the variance. Besides, the single-factor model in CFA did not fit the data as well as the theoretical model, also indicating that the CMB was not serious (Huang, 2012). Altogether, the results from the Harman’s single-factor analysis and the CFA both showed that CMB may not be a substantial problem in this study.

### Descriptive Statistics and Correlations

Means, standard deviations, and correlations of study variables are shown in Table 2. As expected, failure aversion was positively correlated with loss of self-esteem (*r* = 0.21, *p* < 0.01) and loss-focused coping (*r* = 0.08, *p* < 0.05). Meanwhile, loss of self-esteem was negatively correlated with learning from failure (*r* = −0.17, *p* < 0.01). Loss-focused coping was positively correlated with learning from failure (*r* = 0.42, *p* < 0.01), which provided preliminary support for our hypotheses.

**TABLE 2:** Mean, standard deviation, and correlation coefficient among study variables.

	Mean	*SD*	1	2	3	4	5	6	7	8	9
1. Gender	0.77	0.42	−								
2. Age	31.65	5.55	0.04	−							
3. Education level	4.36	0.68	0.00	0.19**	−						
4. Time since last project failure	2.19	0.89	0.09*	0.27**	0.02	−					
5. Failure aversion	3.71	0.95	−0.08*	0.08*	0.06	−0.01	*(0.83)*				
6. Loss of self-esteem	2.64	1.00	0.07	0.01	−0.10**	−0.01	0.21**	*(0.88)*			
7. Loss-focused coping	3.91	0.74	−0.07	0.01	−0.14**	−0.03	0.08*	0.12**	*(0.70)*		
8. Learning goal orientation	4.73	0.70	0.04	−0.07*	−0.08*	0.09*	−0.03	−0.09*	0.17**	*(0.85)*	
9. Learning from failure	4.58	0.84	−0.06	−0.02	−0.040	0.01	−0.02	−0.17**	0.42**	0.35**	*(0.91)*

Coefficient alpha are in parentheses on the diagonal. N = 774, *p < 0.05, **p < 0.01.

### Hypothesis Testing

To examine the proposed dual-pathway model simultaneously (i.e., failure aversion predicts learning from failure through changes in self-esteem and loss-focused coping), we conducted a path analysis using Mplus 7.0. As Table 3 shows, failure aversion was positively associated with loss of self-esteem (*b* = 0.23, *SE* = 0.04, *p* < 0.001) and loss-focused coping (*b* = 0.06, *SE* = 0.03, *p* < 0.05), providing support for Hypotheses 1 and 4. Besides, loss of self-esteem was negatively associated with learning from failure (*b* = −0.18, *SE* = 0.03, *p* < 0.001), while loss-focused coping was positively associated with learning from failure (*b* = 0.51, *SE* = 0.05, *p* < 0.001). Thus, Hypotheses 2 and 5 were supported.

**TABLE 3:** The results of hypothesis test.

	Model 1: Mediation						Model 2: Moderation	
Predictors	Loss of self-esteem		Loss-focused coping		Learning from failure		Learning from failure	
	*Estimate*	*SE*	*Estimate*	*SE*	*Estimate*	*SE*	*Estimate*	*SE*
Gender	0.21*	0.09	−0.11	0.06	−0.03	0.06	−0.05	0.06
Age	0.00	0.01	0.01	0.01	−0.01	0.01	−0.00	0.01
Education	−0.17**	0.05	−0.17***	0.04	0.02	0.05	0.03	0.04
Time since project failure	−0.03	0.04	−0.01	0.03	0.03	0.03	−0.00	0.03
Failure aversion	0.23***	0.04	0.06*	0.03				
Loss of self-esteem (LSE)					−0.18***	0.03	−0.56**	0.20
Loss-focused coping (LCS)					0.51***	0.05	0.76**	0.29
Learning goal orientation (LGO)							0.34	0.29
LSE×LGO							0.09*	0.04
LCS×LGO							−0.07	0.06

The above estimates represent unstandardized path coefficients. N = 745, *p < 0.05, **p < 0.01, ***p < 0.001.

To examine the mediating roles of loss of self-esteem and loss-focused coping, we employed bootstrapping to estimate the indirect confidence interval based on 10,000 simulated samples. Our results showed that the indirect effect of failure aversion on learning from failure through loss of self-esteem was significant (*b* = −0.04, *SE* = 0.01, *p* < 0.001*;* 95% CI [−0.06, −0.03]). Similarly, the indirect effect of failure aversion on learning from failure through loss-focused coping was also significant (*b* = 0.03, *SE* = 0.01, *p* < 0.05*;* 95% CI [0.01, 0.06]). Thus, Hypotheses 3 and 6 were supported.

Hypotheses 7 and 8 proposed the moderating role of LGO. As shown in Table 3, the interactive term of loss of self-esteem and LGO was significantly and positively associated with learning from failure (*b* = 0.09, *SE* = 0.04, *p* < 0.05). However, the coefficient of the interactive term of loss-focused coping and LGO was not significant (*b* = −0.07, *SE* = 0.06, *p* = *n.s.*). Thus, Hypothesis 7 was supported and Hypothesis 8 was not supported by our data.

**FIGURE 2:**
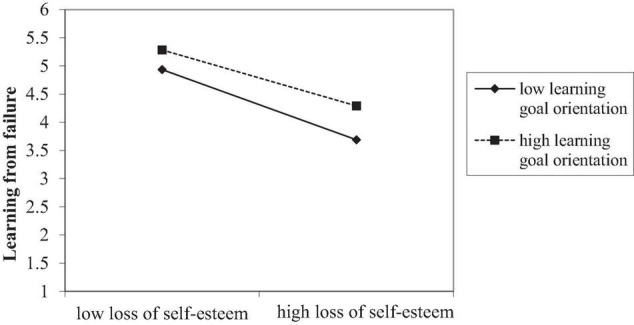
The moderating effect of learning goal orientation.

Following Cohen and Cohen (1983) recommendation, we plotted this moderating effect on the conditional values of LGO (mean ± 1 SD). As shown in Figure 2, the negative relationship between loss of self-esteem and learning from failure was stronger when LGO was at a lower level (*simple slope* = −0.62, *SE* = 0.23, *p* < 0.01), while this relationship became weaker for the individuals with higher levels of LGO (*simple slope* = −0.50, *SE* = 0.17, *p* < 0.01).

### Supplementary Analyses

To extend Hypothesis 7, we further examined whether LGO could moderate the indirect effect of failure aversion on learning from failure via loss of self-esteem (i.e., the moderated mediation effect). Following Edwards and Lambert (2007), we estimated this indirect effect at higher (+1 *SD*) and lower (−1 *SD*) levels of LGO. Our results show that the indirect effect was −0.11 with a 95% CI [−0.19, −0.05] for employees with higher levels of LGO, while the indirect effect was −0.13 with a 95% CI [−0.24, −0.06] for employees with lower levels of LGO. The estimate of the difference between these two indirect effects was 0.025 with 95% CI [0.01, 0.05], indicating that LGO moderated the indirect effect of failure aversion on learning from failure via loss of self-esteem.

## Discussion

Using a sample of 774 employees from R&D teams, our findings showed that individuals’ failure aversion influences their learning from failure through the arguing process. Specifically, it enhanced learning from failure through inducing loss-focused coping, whereas it impeded learning from failure through increased loss of self-esteem. In addition to the arguing process, we further examined the expecting process and choose LGO as an important moderator. We found that individuals’ LGO can moderate the relationship between the arguing process and learning from failure. In other words, the negative relationship between the loss of self-esteem and learning from failure was weaker for employees with higher LGO. Besides, LGO also can mitigate the detrimental indirect effect of failure aversion on learning from failure via loss of self-esteem (i.e., a moderated mediation effect).

In contrast to our hypothesis, loss-focused coping was positively related to learning from failure, irrespective of the level of LGO. We speculate that a threshold may exist in the rate of arguing/cognitive processing. Below the threshold, more cognitive resources can lead to more desirable outcomes. Above the threshold, additional cognitive resources can only lead to a fixed (maximum) processing rate, which means that additional resources cannot further facilitate desirable outcomes. In this study, both loss-focused coping and LGO can provide individuals additional cognitive resources to learn. If individuals who adopt loss-focused coping allocate attentional resources which reach the threshold, the additional resources provided by their LGO cannot further facilitate the individual learning process. Therefore, the moderating effect was not significant.

### Theoretical Implications

The current study contributes to the literature on learning from failure in three ways. First, we extend this literature by exploring and examining individuals’ attitudes toward failure through the lens of sensemaking theory. Specially, we introduce individuals’ failure aversion as a more fundamental and proximal predictor which can help individuals to notice and analyze failure events from a particular perspective. Although the important role of the attitude toward failures in influencing learning behaviors has been discussed in other fields such as educational psychology and behavioral economics (e.g., Yechiam and Hochman, 2013; Alamri and Fawzi, 2016), rarely have organizational scholars paid attention to the role of individuals’ attitude toward failures in predicting their subsequent learning behaviors. Van Dyck et al. (2005) aggregated individual-level failure aversion to the level of organizational failure aversion, positing it as part of the organizational culture, and examined its impacts on firm performance. However, such an approach cannot address the fact that the attitude toward failure varies with individuals. For example, employees can still interpret failure and respond to it negatively, irrespective of whether an organizational failure aversion culture exists or not. Based on the sensemaking theory, our study bridges this theoretical gap by examining individuals’ failure aversion in the project failure context.

Second, we reveal the mechanism of how failure aversion would influence learning from failure through a cognitive perspective. Although Frese and Keith (2015) have suggested that failures can trigger different cognitive responses, rarely have researchers examined and developed this assumption. Based on the sensemaking theory, when people notice the interrupted event, they will construct meanings through a process of positing and evaluating arguments. During this process, individuals will generate different cognitions (including positive and negative framing) at the same time, which exert different effects on the learning from failure process. We found that failure aversion reduced or enhanced subsequent learning activities through making changes in self-esteem and loss-focused coping, respectively. Thus, our study provides empirical evidence for how failure aversion influences learning from failure through different cognitive mechanisms.

Moreover, previous researchers have shown that individuals’ aversion attitude usually leads to negative outcomes. For example, Fullerton and Umphrey (2016) found that individuals who are math averse have lower mathematics scores. However, we also found a positive influence of failure aversion on individual learning behavior, which encourages future researchers to explore the bright side of an attitude of failure aversion.

Finally, we contribute to the literature on learning from failure by considering the role of LGO in the relationship between cognitive reactions and learning behavior. Although the important role of LGO has been acknowledged in learning domains, few researchers have paid attention to its role in learning from failure in the work context. This might be because the direct effect between LGO and learning from failure was not supported in a previous study (Zhao, 2011). Based on the sensemaking theory, our findings show that LGO might not contribute to learning from failure directly; instead, it moderates the arguing process after failure. As an important factor that can determine individuals’ expectations toward learning from failure, LGO can buffer the negative relationship between negative cognitive reactions (i.e., the loss of self-esteem) and learning behaviors. Thus, our study empirically supports the important moderating role of LGO in influencing individuals’ cognitive resources to learn.

### Practical Implications

Our findings also have several practical implications. First, our findings can both benefit the institutions of higher education and organization. Our results showed that individuals’ attitude toward failure (i.e., failure aversion) is an important factor that can determine their subsequent behavior toward failure (i.e., learning from failure). Thus, higher education or organization should cultivate students/employees’ correct attitude toward failure to let them treat failure as a self-improvement opportunity rather than criticizing themselves and being negative about themselves when experiencing failures.

Second, our study also reveals that failure aversion can both impede and facilitate individual learning from failure through two different cognitive mechanisms. One is to trigger individual negative evaluation cycles (i.e., a loss of self-esteem); another is to lead individuals to allocate more cognitive resources to the pool of failure events to deal with them (i.e., loss-focused coping). These findings suggest that individuals can learn more from failure by rebuilding their self-esteem or developing their loss-focused coping. For rebuilding and maintaining individual self-esteem, managers should provide numerous supports (i.e., authorization and permission) for individuals. Researchers have shown that a supportive environment can promote individual self-esteem (Raboteg-Saric and Sakic, 2014). To develop individuals’ loss-focused coping, managers can attempt to reduce employees’ negative emotional responses associated with failure by acknowledging employees’ abilities to restore their confidence or by letting employees talk about their feelings to relieve their negative emotions. On the other hand, managers can remove or reduce the cause of the stressors by providing some problem-solving methods or resources.

Third, our findings show that LGO can buffer the negative relationship between a loss of self-esteem and learning from failure. Accordingly, apart from managing individual cognitive reactions after failures through providing numerous supports, managers can also select or cultivate individuals’ LGO to promote their learning abilities under failure aversion. For selecting employees with higher LGO, managers can use the LGO measurement scale to screen people. For cultivating employees’ LGO, managers can provide a learning opportunity, give positive feedback on their employees’ learning activities, or set specific learning goals for their employees to cultivate their LGO (Bezuijen et al., 2009).

### Limitations and Future Research Directions

In addition to the above contributions, we acknowledge that our study also has some limitations. The first relates to the use of self-report as the single point to measure the constructs for our model. However, we believe that this research design is adequate for the following reasons. First, individual attitudinal evaluations, cognitive experience, and behavioral ratings are best reported by the individuals themselves (Berry et al., 2012). Second, our questionnaire measured relatively stable variables (i.e., failure aversion and LGO) in the former part, and then we asked participants to recall their cognitive responses and their learning behaviors after a recent failure. This procedure can separate the variables that occur at different time points (i.e., before and after failure). Finally, the results of the CFA and Harman’s test showed that CMB may not be a substantial problem in this study. Nevertheless, multiple data sources (i.e., leaders or colleagues) at multiple points are recommended in future research to reduce the potential for CMB.

Second, although we have used the sensemaking theory to develop our model explaining how individuals’ failure aversion would influence their learning from failure through arguing and considering the expectation process, we did not measure individuals’ sensemaking perception directly. Thus, we recommend future research explicitly measure individuals’ sensemaking perception, thereby providing more robust evidence.

Third, our study only used a quantitative method to assess individual cognitive and behavioral responses after failure. Although the quantitative method can capture individual cognitive and behavioral responses, asking participants to recall their failure experience may lead to biases due to their need for social desirability and their cognitive state. Thus, we recommend using mixed methods in future research. For instance, researchers can use a case study to explore the deeper underlying mechanisms of the attitude-behavior relationship.

## Data Availability Statement

The raw data supporting the conclusions of this article will be made available by the authors, without undue reservation.

## Ethics Statement

The studies involving human participants were reviewed and approved by the Human Research Ethics Committee (HREC) at the Beijing Normal University. The patients/participants provided their written informed consent to participate in this study.

## Author Contributions

LZ, BW, and WW performed the data collection and analysis. LZ and XF wrote the first draft of the manuscript. All authors contributed to the study conception and design, commented on previous versions of the manuscript, and read and approved the final manuscript.

## Conflict of Interest

The authors declare that the research was conducted in the absence of any commercial or financial relationships that could be construed as a potential conflict of interest.

## Publisher’s Note

All claims expressed in this article are solely those of the authors and do not necessarily represent those of their affiliated organizations, or those of the publisher, the editors and the reviewers. Any product that may be evaluated in this article, or claim that may be made by its manufacturer, is not guaranteed or endorsed by the publisher.
